# Characterization of *Phytopythium* Species Involved in the Establishment and Development of Kiwifruit Vine Decline Syndrome

**DOI:** 10.3390/microorganisms11010216

**Published:** 2023-01-15

**Authors:** Simona Prencipe, Giada Schiavon, Marco Rosati, Luca Nari, Leonardo Schena, Davide Spadaro

**Affiliations:** 1 Department of Agricultural, Forestry and Food Sciences (DiSAFA), University of Torino, Via Paolo Braccini 2, 10095 Grugliasco, Italy; 2 Centre of Competence for the Innovation in the Agro-Environmental Sector—AGROINNOVA, University of Turin, Via Paolo Braccini 2, 10095 Grugliasco, Italy; 3 Fondazione Agrion, Via Falicetto, 24, 12030 Manta, Italy; 4 Dipartimento di AGRARIA, Università Mediterranea di Reggio Calabria, Feo di Vito, 89122 Reggio Calabria, Italy

**Keywords:** oomycete, kiwifruit, cardinal temperature, pH, multi-locus sequence typing, morphology, pathogenicity, Italy

## Abstract

Since 2012, the kiwifruit vine decline syndrome (KVDS) has progressively compromised Italian kiwifruit orchards. Different abiotic and biotic factors have been associated with the establishment and development of KVDS. During monitoring of orchards affected by KVDS in north-western Italy during 2016–2019, 71 *Phytopythium* spp. were isolated. Based on maximum likelihood concatenated phylogeny on the ITS1-5.8S-ITS2 region of the rDNA, large subunit rDNA, and cytochrome oxidase I, isolates were identified as *P. vexans* (52), *P. litorale* (10), *P. chamaehyphon* (7) and *P. helicoides* (2). *Phytopythium litorale* and *P. helicoides* are reported for the first time as agents of KVDS in Italy. To demonstrate pathogenicity and fulfil Koch’s postulates, representative isolates of *P. vexans*, *P. litorale*, *P. chamaehyphon* and *P. helicoides* were inoculated in potted plants. In these trials, waterlogging was applied to stress plant with a temporary anoxia and to favour the production of infective zoospores by the oomycetes. In experiments in vitro, the four species showed the highest growth at 25–30 °C, depending on the media used. *P. helicoides* was able to grow also at 40 °C. The four species were able to grow in vitro at a pH ranging from 5.0 to 8.0, showing that pH had less effect on growth than temperature. The present study suggests a strong role of different species of *Phytopythium* in the establishment and development of KVDS. *Phytopythium* spp. could be favoured by the average increase in soil temperatures during summer, associated with global warming.

## 1. Introduction

Kiwifruit production is around 3.5 million tons worldwide [1], and Italy is the second kiwifruit producer after China, with 316,443 tons in 2019, whose 250,000 tons are exported [2]. Since 2012, kiwifruit vine decline syndrome (KVDS) has affected over 10% (almost 2900 ha) of Italian kiwifruit orchards, in various regions of northern (Veneto, Piedmont, Friuli Venezia Giulia), central (Lazio, Emilia Romagna) and southern Italy (Calabria) [1]. However, it is estimated that over 25% of the Italian kiwifruit orchards are compromised [3]. In the areas where KVDS is observed, the most common irrigation system consists of temporary flooding [4,5].

Up to now, the syndrome has been reported only in Italy. Similar vine decline disorders were reported also in other countries but were attributed to a specific pathogen [6,7], to waterlogging [8], to root rotting, or to root asphyxia [9]. 

Typical KVDS symptoms are a reduction in plant vigour, progressive leaf desiccation starting from the margin to the centre of the leaves, leaf curling with a progression from the basal to the upper leaves [3,10]. Canopy symptoms are generally related to damage of structural roots with brown rotting areas and absence of feeder roots, even though vines with compromised roots could still have asymptomatic canopy [11]. The symptomatology is more evident during summer, when high temperatures and transpiration are recorded. Once the syndrome becomes visible, the plant dies within one year [12].

Different research has aimed to understand the aetiology of KVDS, suggesting that different abiotic and biotic factors may be involved. Sorrenti et al. [1] reported a high disease frequency in silty-sandy soil, where temporary waterlogging occurs. Soil drainage seems to represent one important factor involved in the syndrome. Waterlogging could promote KVDS, even though the disease is reported also on sandy or well-drained soils [3,5,13,14]. Soil structure and its management are also involved in KVDS development [15], as well as the interaction between these factors and weather conditions [12]. As reported by Tacconi et al. [16], a delay in the development of KVDS was obtained using soil convexing and compost in order to improve water drainage and soil texture.

The involvement of biotic factors in KVDS was demonstrated by Savian et al. [3], where symptom development was obtained using soil from infected KVDS orchards, whereas no symptoms were observed using sterilised soil. Different pathogens have been associated with KVDS. Among fungal species isolated from symptomatic tissues, the pathogenicity on kiwifruits plants was demonstrated for *Phytophthora cryptogea*, *P. citrophthora*, *Phytopythium vexans*, *P. chamaehyphon*, and *Desarmillaria tabescens* [5,10,11,17]. Among bacteria, the genus *Clostridium* was associated with the disease [18] even though Donati et al. [11] did not isolate bacterial pathogens neither from affected KVDS orchards nor from healthy ones.

The aim of the present work was to investigate the presence of microorganisms associated with KVDS and their role in the syndrome development, with a focus on oomycetes. The strains were identified by morphological and molecular approaches. Biological characterisation of the isolates included pathogenicity tests performed to reproduce typical symptoms of KVDS and the evaluation of the effect of temperature and pH on their growth.

## 2. Materials and Methods

### 2.1. Oomycete Isolation

*Phytopythium* spp. isolates were collected from 18 *A. chinensis* orchards during the period August–October of 2016, 2018 and 2019, in Piedmont, north-western Italy (Table 1 and Table S1). The strains were isolated from kiwifruit plants showing typical KVDS symptoms, i.e., reduction in plant vigour, leaf curling, or complete decline, focusing on microorganisms associated with infected tissues. Isolation was carried out from symptomatic rotten roots at the margin between infected and healthy tissue to fulfil the first postulate of Koch, as previously described by Prencipe et al. [10]. Briefly, roots were first surface-disinfected with 1% sodium hypochlorite, washed in sterile deionised water and air-dried. Five fragments of each root were cut at the symptom edges and plated onto Potato Dextrose Agar (PDA, Merck, Germany) and semi-selective oomycete PARP medium (17 g corn meal agar, 0.01 g Pimaricin, 0.01 g Ampicillin 0.01 g, Rifampicin and 0.07 g Pentachloronitrobenzene,) Petri dishes. After 4 days of incubation at 25 ± 1 °C, 71 representative isolates were selected (Table 1), based on colony morphology, and they were maintained in tubes of PARP medium.

### 2.2. Molecular Identification

Genomic DNA was extracted from isolates grown in Potato dextrose broth (PDB, Merck, Darmstadt, Germany) at 25 ± 1 °C on rotary shaker for 8 days, using an Omega E.Z.N.A. Fungal DNA Mini Kit (VWR, Radnor, USA), according to manufacturer’s instructions. The ITS1-5.8S-ITS2 region of the rDNA was amplified using primers ITS1 and ITS4 or ITS4 and ITS6 and protocols reported in White et al. [19] and Cooke and Duncan [20]. The large subunit (LSU) rDNA, was amplified using primers (NL1/NL4) and protocol reported in Baten et al. [21] Finally the cytochrome oxidase I gene (COI) was amplified using primes (FM85mod/OomCOILevup) and protocol reported in Robideau et al. [22]. PCR were carried in a volume of 25 µL using: 2.5 µL of Qiagen PCR Buffer, 0.5 µL of MgCl_2_, 0.75 µL of dNTPs (10 mM), 1 µL of each primer (10 µM), 0.2 µL of Taq DNA polymerase and 1 µL (20 ng) of template DNA. The amplicons were checked by electrophoresis at 110 V/cm for 40 min in 1% agarose gel stained with 1 µL of GelRed™ (VWR). Single PCR fragments were purified using QIAquick© PCR purification Kit (Qiagen, Hilden, Germany), and sequenced in both directions by Macrogen, Inc. (Amsterdam, The Netherlands). The DNA Baser program (Heracle Biosoft S.R.L., Arges, Romania) was used to obtain the consensus sequences and alignment was performed using CLUSTALW through Molecular Evolutionary Genetics Analysis (MEGA6) software, version 6.0. After cutting the trimmed regions and manual correction, a dataset of 761 bp, 723 bp and 658 bp for ITS, LSU and COI, respectively, was obtained. The best-fit nucleotide model for the concatenated dataset was obtained using MEGA version 6, as well as to perform the phylogenetic analysis with the Maximum likelihood (ML) algorithm. Reference sequences used for phylogeny, according to the last revision of the genus [23] and the latest species descriptions, are reported in Table S2. All sequences were deposited in GenBank (Table 1).

### 2.3. Morphological Observation

For the macro-morphology, two representative isolates per species (Table 1) were grown onto PDA, Corn Meal Agar (CMA) and Potato Carrot Agar (PCA) Petri dishes [24], as described by de Cock & Lévesque [25]. Each plate was inoculated with a 6 mm mycelial plug in the centre of the plate and observed for colony characteristics (growth, colour, margin shape and texture), after 5 days of incubation at 25 ± 1 °C in the dark.

For the micro-morphology, sporangia and zoospore productions were induced for two isolates per species (Table 1), as described by de Cock & Lévesque [25]. Briefly, pieces of sterilised grass blades were placed onto CMA Petri dishes colonised by actively growing mycelium of the oomycetes. After 24 h, grass blades were transferred into Petri dishes filled with 10 mL of sterile soil broth (100 g sandy soil, 1 L deionised water), incubated at 20 ± 1 °C and exposed to 16 h daylight. Observations were carried out using a Nikon (Eclipse 55t) microscope (Tokyo, Japan) at 40× magnification after 7–14 days, depending on the strain. Twenty measurements were made for each isolate.

### 2.4. Effect of Temperature and pH on In Vitro Growth

For the assessment of the growth cardinal temperatures, the same isolates used for morphological observation (Table 1) were inoculated onto PDA, PCA and CMA, and incubated at 10, 15, 20, 25, 30, 35, 40 and 45 ± 1 °C for 5 days according to de Cock & Lévesque [25] and de Cock et al. [23]. Radial growth was measured daily, along two lines intersecting the centre of the plate, where the inoculum plug (6 mm) was positioned, and data were expressed as growth rate (mm/day). 

To assess the effect of pH on the growth of colonies, a mycelial plug (6 mm) of each isolate was inoculated on three plates containing PCA adjusted at specific pH and incubated at 25 ± 1 °C for 5 days. To obtain PCA at specific pH (5.0, 5.5, 6.0, 6.5, 7.0, 7.5, and 8.0), NaoH 1M and HCl 1M solutions were used. The pH was measured using FiveEasy pH meter (Mettler Toledo, Milano, Italy). Radial growth was measured daily along two lines intersecting the centre of the mycelial plug, and data were expressed as growth rate (mm/day). 

### 2.5. Pathogenicity Test

**Figure 1:**
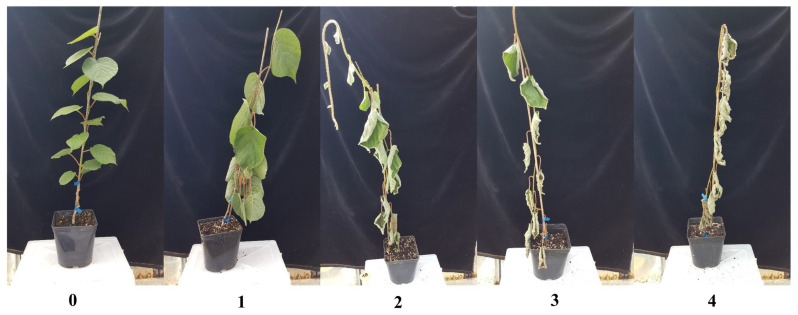
Disease severity index used to evaluate in greenhouse the pathogenicity of the *Phytopythium* species inoculated on 1-year-old *A. chinensis* var. *deliciosa* ‘Hayward’ plants.

The pathogenicity test was carried out on 1-year-old plants of *A. chinensis* var. *deliciosa* ‘Hayward’ grown in 3 L pots containing a sterilised agriperlite-peat mixture. One representative isolate strain per species was used for the inoculation of five kiwifruit plants: *P. vexans* strain PP1, *P. chamaehyphon* strain PH6, *P. helicoides* strain CA2 and *P. litorale* strain R3A. The inoculum was prepared according to Prencipe et al. [10]. Briefly, each strain was grown for 7 days on a wheat and hemp mixture (100 g wheat, 50 g hemp and 170 mL of water), and used to inoculate the soil at a rate of 6 g/L per pot. Similarly, negative control plants were inoculated with a sterile seed mixture, whereas positive control plants were potted onto diseased-infected soil taken from an orchard of *A. chinensis* var. *deliciosa* ‘Hayward’ located in Saluzzo (Piedmont, north-western Italy), showing typical KVDS symptoms. Plants were kept in greenhouse at 28 ± 5 °C. In order to simulate the field capacity, 2 days post inoculation (dpi), all of the plants were submerged up to the crown level for 72 h. Based on symptoms observed, the disease severity (DS) on *Actinidia* plants was scored at 12 and 26 dpi using a scale of 0 to 4 (Figure 1), corresponding to: 0 = healthy plant; 1 = plant starting to wither at the basal level; 2 = plant showing withering to the upper level and basal leaves showing curling; 3 = basal leaves dried and upper leaves curling; 4 = dead plant. 

To fulfil Koch’s postulates, re-isolations were performed from the roots of all the plants and the isolates were identified by sequencing the COI gene, as described above. Furthermore, soil pH and temperature were measured at the beginning of the trial and at 26 dpi with a pH-meter and a temperature probe. After 26 dpi, the dry weight of the roots was also measured.

### 2.6. Statistical Analysis

Statistical analyses on the in vitro test and the pathogenicity test were carried out by using IBM SPSS (Chicago, IL, USA) statistics version 25 for the normal distribution analysis using Shapiro-Wilk normality test, the homogeneity of variance using Levene’s test and one-way analysis of variance using Tukey’s test (*p* ≤ 0.05). 

## 3. Results

### 3.1. Oomycete Isolation and Identification

During the summer-autumn period of three years (2016, 2018 and 2019), several isolates of *Phytopythium* spp. were collected from plants of *A. chinensis* showing symptoms of KVDS. Based on colony morphology 26, 12, and 33 representative isolates were selected in 2016, 2018, and 2019, respectively, to obtain a total of 71 isolates (Table 1). The isolates collected in 2016 were all from ‘Hayward’ kiwifruit, except for one isolate from the cultivar ‘Soreli’. The isolates collected in 2018 were from ‘Hayward’ plants, while those collected in 2019 were from different cultivars: twenty-three from ‘Hayward’, a green pulp variety, eight from ‘Soreli’, a yellow pulp variety, and two from ‘Dong Hong’, a red pulp variety. 

**Figure 2:**
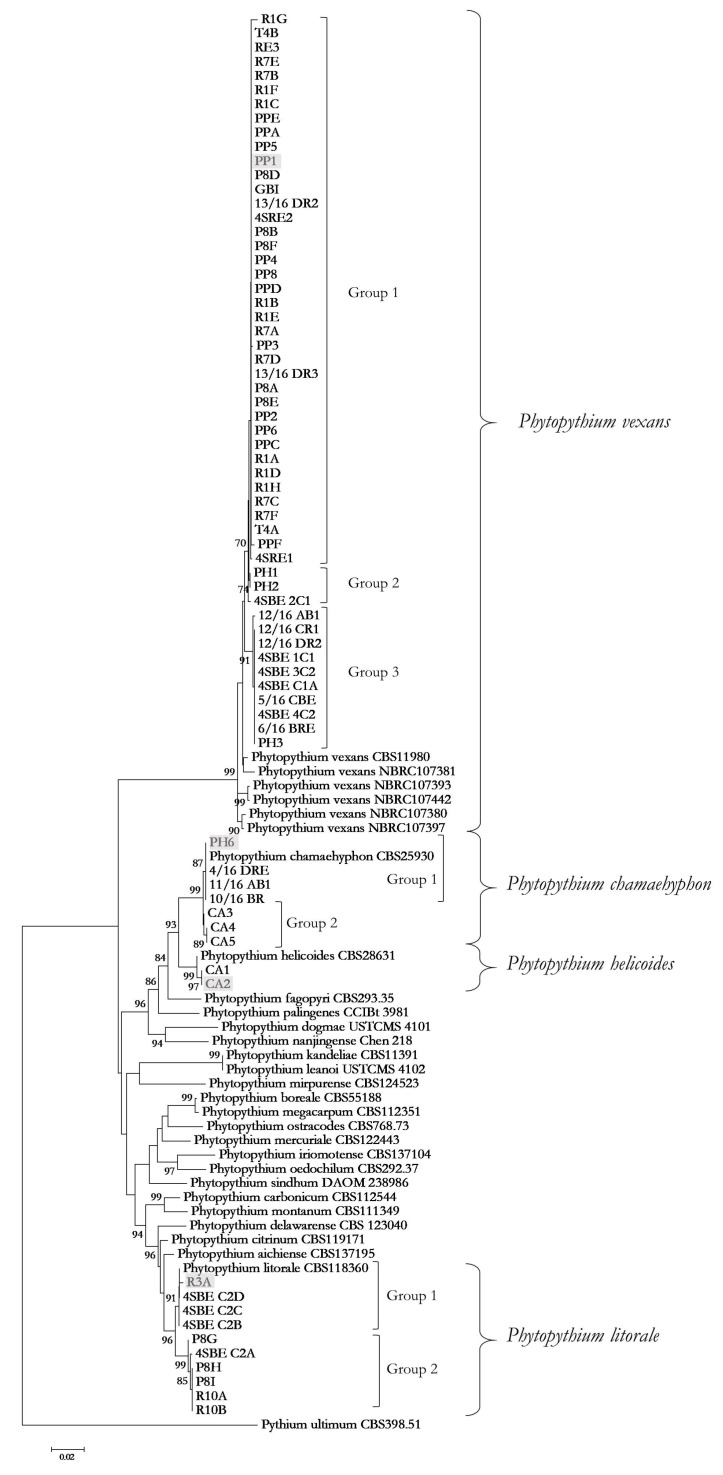
Best scoring Maximum Likelihood tree based on the concatenated ITS, LSU, and COI sequence datasets. The numbers at the major nodes indicate the bootstrap value from 1000 bootstrapped datasets. Branches with lower bootstrap values than 70% are not shown. Phylogeny was rooted by *Pythium ultimum*. Evolutionary analyses were conducted using MEGA, version 6. The highlighted strains are the isolates used for pathogenicity test.

Sequencing of the ITS1-5.8S-ITS2 region, the large subunit (LSU) rDNA, and cytochrome oxidase I gene (COI) were used for species assignation. The best-fit model used for the concatenated dataset was Tamura Nei + Gamma distribution. Based on concatenated phylogeny, 52 isolates were identified as *P. vexans*, 10 as *P. litorale*, seven as *P. chamaehyphon*, and two as *P. helicoides* (Figure 2). 

A great intraspecific variability was observed. *P. vexans* strains were divided into three main groups: one with 39 strains, isolated in the three years of sampling, the second group with three strains, two isolated in 2016 and one in 2018, and 10 strains in the last group, all isolated in 2016 (Figure 2, Table 1). *P. chamaehyphon* strains clustered into two groups: one with four strains isolated during 2016 and the second group with three strains isolated in 2019 (Figure 2, Table 1). *P. litorale* strains were also divided into two groups: the first comprises three strains isolated in 2016 and one in 2019, and the second group has one strain isolated in 2016 and five strains isolated during 2019 (Figure 2, Table 1).

In 2016, 10 orchards were sampled (Table S1) and enabled the isolation of 26 strains: 18 identified as *P. vexans*, four as *P. litorale* and four as *P. chamaehyphon*. *P. vexans* was isolated in all the orchards while *P. chamaehyphon* and *P. litorale* were isolated only in orchards 1 and 2, respectively. In 2018, all isolates (12) were identified as *P. vexans*, whereas in 2019 the sampling in eight orchards (Table S1) yielded 22 strains: 11 identified as *P. vexans*, six as *P. litorale*, three as *P. chamaehyphon*, and 2 as *P. helicoides*. Only *P. vexans* was isolated from six orchards sampled in 2019. In orchard 12 both *P. chamaehyphon* and *P. helicoides* were isolated, whereas in orchard 16 both *P. vexans* and *P. litorale* were found.

### 3.2. Morphological Observations 

For the macro-morphology, two representative isolates per species were grown onto PDA, CMA and PCA Petri dishes and observed after 5 days. The same two strains were also grown on soil broth containing grass blades and their micro-morphology was observed under microscope after 7–14 days.

**Figure 3:**
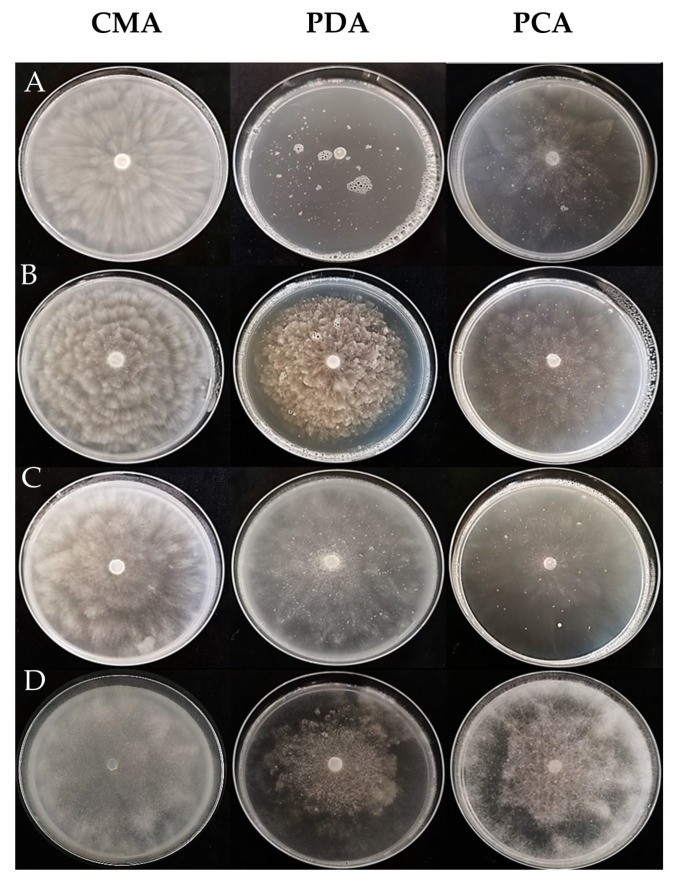
Colony morphology of *Phytopythium* species isolated from kiwifruits on Corn Meal Agar (CMA), Potato Dextrose Agar (PDA) and Potato Carrot Agar (PCA) after 6 days at 25 ± 1 °C. (**A**) *Phytopythium vexans;* (**B**) *Phytopythium litorale*; (**C**) *Phytopythium chamaehyphon*; (**D***) Phytopythium helicoides*.

*Phytopythium vexans* colonies showed aerial mycelium and radiate chrysanthemum mycelial pattern on CMA (Figure 3A; mean diameter: 78 mm), a submerged mycelium without visible pattern on PDA (Figure 3A; mean diameter: 46 mm), and a submerged mycelium and a slight radiate chrysanthemum pattern on PCA (Figure 3A; mean diameter: 79 mm). Hyphae were hyaline, 7 to 15.19 μm wide. Sporangia were subglobose (20.17 ± 3.56 μm × 19.59 ± 3.26 μm) non-papillate. Oogonia were not produced. No differences between the two strains were observed.

*Phytopythium litorale* colonies showed aerial mycelium and rosette mycelial pattern on CMA and PDA (Figure 3B; mean diameter of 79 and 63 mm, respectively), whereas submerged mycelium and radiate chrysanthemum pattern on PCA (Figure 3B; mean diameter: 79 mm). Hyphae were hyaline, 6 to 15.95 μm wide. Sporangia and oogonia were not produced. No differences between the two strains were observed.

*Phytopythium chamaehyphon* colonies showed aerial mycelium and a radiate chrysanthemum pattern on CMA and PDA (Figure 3C; mean diameter: 79 mm), whereas submerged mycelium and radiate chrysanthemum pattern on PCA (Figure 3C; mean diameter: 79 mm). Hyphae were hyaline, 2.29 to 5.13 µm wide. Sporangia were subglobose (25.80 ± 2.60 μm × 25.73 ± 3.69 µm) non-papillate. Oogonia were not produced.

*Phytopythium helicoides* colonies showed aerial mycelium and no specific pattern on CMA, PDA and PCA (Figure 3D; mean diameter: 79 mm). Hyphae were hyaline, 8.49 to 13.42 μm wide. Sporangia were globose or ovoid (36.93 ± 6.78 μm × 39.04 ± 7.96 μm) mainly without papilla, and oogonia were smooth and spherical (27.78 ± 4.28 × 26.06 μm ± 2.96 μm). No differences between the two strains were observed.

### 3.3. Effect of Temperature and pH on In Vitro Growth

**Figure 4:**
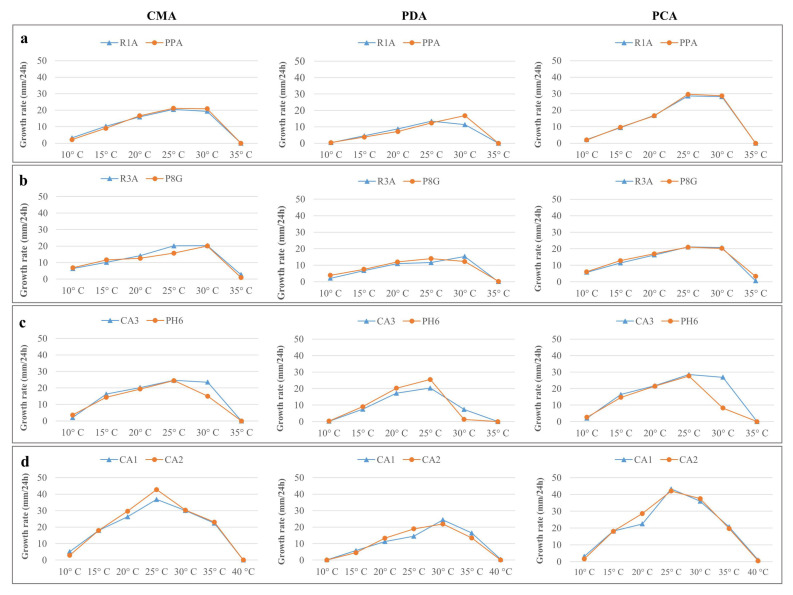
Average radial growth rate (mm/24 h) of *Phytopythium* species grown onto CMA, PDA and PCA media at different temperatures for 5 days. (**a**) *P. vexans* strains R1A and PPA; (**b**) *P. litorale* strains R3A and P8G; (**c**) *P. chamaehyphon* strains CA3 and PH6; (**d**) *P. helicoides* strains CA1 and CA2.

The optimal growth for *P. vexans* strains occurred at 25 °C on CMA and PCA, with an average radial growth of 21 and 29 mm/24 h, respectively (Figure 4a). Onto PDA, it was 25 °C for the strain R1A, with an average radial growth of 13 mm/24 h, whereas it was at 30 °C for the strain PPA, with an average radial growth of 17 mm/24 h (Figure 4a). The minimum and maximum growth temperature were 10 °C and 30 °C, respectively, on all media. 

The optimal growth for *P. litorale* strains occurred at 30 °C on CMA, with an average radial growth of 20 mm/24 h (Figure 4b). Onto PDA, it was at 25 °C for the strain R3A, with an average radial growth of 12 mm/24 h, and at 30 °C for the strain P8G, with an average radial growth of 12 mm/24 h. Onto PCA, for both strains, it was at 25 °C with an average radial growth of 21 mm/24 h. The minimum and maximum growth temperature were 10 °C and 35 °C, respectively, on all media.

The optimal growth for *P. chamaehyphon* strains occurred at 25 °C on CMA, PDA and PCA, with an average radial growth of 25, 23, and 23 mm/24 h, respectively (Figure 4c). The minimum and maximum growth temperature were 10 °C and 30 °C, respectively, on all media. 

The optimal growth for *P. helicoides* strains occurred at 25 °C on CMA and PCA media with an average radial growth of 40 mm/24 h and 42 mm/24 h, respectively (Figure 4d). Onto PDA media was 30 °C, with an average radial growth of 23 mm/24 h. The minimum temperature for growth was 10 °C in all the media, whereas the maximum growth temperatures was 35 °C on PDA and 40 °C on CMA and PCA. 

**Figure 5:**
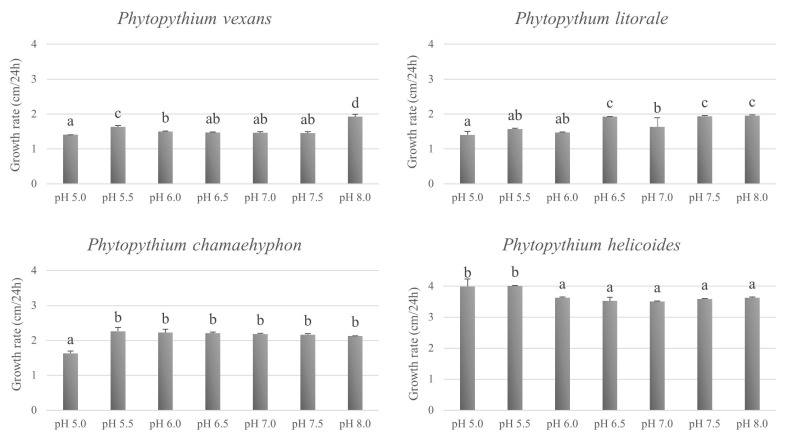
Average radial growth rate (cm/24 h) of two strains per *Phytopythium* species grown onto PCA media adjusted at specific pH at 25 °C for 5 days. Values are expressed as mean values ± SD (n = 6). Values followed by the same letter are not statistically different by Tukey’s test (*p* ≤ 0.05).

The four species tested were able to grow on PCA at pH from 5.0 to 8.0, with different growth rate (Figure 5). Since no statistical different growth rate (*p* ≥ 0.05) was found for both strains of the same species, the values shown are the mean of the two strains. *P. vexans* showed the highest growth rate at pH 8.0 and pH 5.5 (*p* ≤ 0.05), whereas *P. litorale* at pH from pH 6.5 to 8.0 (*p* ≤ 0.05). For *P. chamaehyphon*, there was no statistical different growth rate from pH 5.5 to 8.0, whereas a lower growth rate was shown at pH 5.0 (*p* ≤ 0.05). The optimal pH was between pH 5.0 and 5.5 (*p* ≤ 0.05) for *P. helicoides*.

### 3.4. Pathogenicity Test

All *Phytopythium* species under investigation were able to induce leaf curling, root rot, and decline of inoculated *Actinidia* plants. The first symptoms occurred after 12 days post-inoculation (Table 2) in all of the inoculated plants, whereas negative controls remained symptomless. *P. helicoides* showed the highest disease index (3.67 ± 0.58) and no statistical differences were observed when compared to the positive control (infected soil). The other species showed a slow progression of symptoms (Table 2). After 26 dpi, the species with the highest virulence remained *P. helicoides* (4.00 ± 0.00) compared to the other species tested, and no statistical differences were observed when compared to the positive control (Table 2). *P. vexans* and *P. chamaehyphon* showed similar virulence, with a mean disease index of 2.17 ± 0.58 and 2.67 ± 0.29, respectively. These species showed a lower disease index and the disease progressed more slowly during the test. *P. litorale* showed to be more virulent (3.17 ± 0.76) compared to *P. vexans*, but no statistical differences were observed compared to the positive control (Table 2).

To fulfil Koch’s postulates, re-isolations were performed from the roots of all the plants and the isolates were identified as *P. vexans*, *P. helicoides*, *P. chamaehyphon* and *P. litorale*.

The soil pH at the beginning of the trial was 4.35 ± 0.22, whereas after 26 dpi it was 5.33 ± 0.22. The soil temperature was 23.74 ± 0.76 °C when the trial started and it was 22.06 ± 0.81 at the end of the trial. The highest dry weight was recorded from roots sampled from healthy plants (40.05 ± 8.32 g) compared to the other roots (Table 3). No statistical differences were observed between roots inoculated with *P. vexans*, *P. litorale* and *P. chamaehyphon* (*p* ≤ 0.05), whereas a statistically lower dry weight was observed for the roots of plants inoculated with *P. helicoides*. No statistical differences were observed when *P. helicoides* was compared to the positive control. 

## 4. Discussion

Field surveys carried out in 18 *A. chinensis* orchards of north-western Italy during 2016–2019 permitted us to collect 71 isolates of *Phytopythium* spp. from kiwifruit plants, showing typical symptoms of KVDS, such as reduction in plant vigour, leaf curling, or complete decline. Isolation from roots of plants affected by KVDS is a difficult process, as it involves root surface disinfection, washing, air-drying, tissue sample taking from the affected area, plating on PDA and oomycete PARP media and, after 4 days, transplant into tubes with PARP medium. All of the isolates showed to belong to the oomycete genus *Phytopythium*. 

The role of the microbial community in KVDS development was previously demonstrated. Donati and colleagues [11] underlined the role of the rhizosphere microbial community, since a high incidence of *Phytophthora* spp. and *Phytopythium* spp. was associated with plants affected with KVDS. The role of biotic components in KVDS development was also described by Savian et al. [3], where in greenhouse experiments the symptoms were reproduced using unsterilised soil from a KVDS affected orchard, whereas no symptoms were observed using the same soil sterilised. The most frequently isolated species from kiwifruit affected by KVDS were *Phytophthora citrophthora*, *P. cryptogea*, *P. infestans*, *P. megasperma*, and *Cylindrocarpon* spp. [5,11]. Furthermore, *Phytopythium* spp. were isolated from symptomatic kiwifruits plants in several investigations [5,10,11,17]. 

In the present study, based on ML concatenated phylogeny on the ITS1-5.8S-ITS2 region of the rDNA, the large subunit (LSU) rDNA, and cytochrome oxidase I gene (COI), 52 strains were identified as *P. vexans*, 10 strains as *P. litorale*, seven strains as *P. chamaehyphon* and two strains as *P. helicoides*. Both, *P. litorale* and *P. helicoides* are reported for the first time as agents of KVDS on kiwifruit plants in Italy. Most of the isolates showed to belong to the species *P. vexans*, which is known to cause root and crown root in different crops, including kiwifruit [23,26,27]. Most orchards sampled showed the presence of only one *Phytopythium* species, except for some exceptions where two species were found. 

A great intraspecific variability was observed among the strains of each of the four species, which were divided into clusters. This variability was previously observed for *Phytopythium* species, such as *P. vexans* [21,28,29], *P. helicoides* [21,28,30,31], *P. litorale*, *P. kandeliae* [32], *P. mercuriale* and *P. oedochilum* [33]. Based on micro- and macro-morphology analyses, the selected strains confirmed the typical characteristics of species [25,33,34,35,36].

In the pathogenicity tests, the virulence of all of the isolated species was demonstrated. Previously, *P. vexans* and *P. chamaehyphon* resulted pathogenic on 1-year-old or 6-month-old *Actinidia chinensis* var. *deliciosa* ‘Hayward’ plants [10,17]. Furthermore, the symptoms reproduction was also demonstrated for *Desarmillaria tabescens* and one isolate of *Phytopythium* spp. [11]. In this work, we demonstrated for the first time the pathogenicity of *P. litorale* on kiwifruit plants and *P. helicoides* was reported for the first time on kiwifruit plants in Italy. *P. litorale*, *P. helicoides* and *P. vexans* were previously reported as pathogenic on other hosts, such as on *Platanus orientalis* [37], *Rhododendron pulchrum* [38], on citrus, apple, and pear [39], and on almond [40]. Moreover, *P. helicoides* was already reported as agent of root and collar rot on kiwifruit in China [41]. 

In the pathogenicity test, flooding was used to reproduce soil water content proximal to field capacity. Waterlogging was previously investigated and, when applied alone, it was unable to reproduce KVDS symptoms. However, waterlogging is able to promote the progression of KVDS symptoms [3] and oomycetes were mainly reisolated from plants subjected to high irrigations volumes [11]. It should be noted that *Phytopythium* spp. live in water and soil and need a high humidity to produce sporangia and zoospores that are important infective propagules [42]. Therefore, the presence of water seems an important factor to promote *Phytopythium* spp. propagules and the onset of the disease.

In experiments in vitro, the four species showed an optimal growth at a temperature between 25 and 30 °C, depending on the media used. The maximum growth temperature was 30 °C for *P. vexans*, *P. chamaehyphon*, and *P. litorale.* Only *P. helicoides* was able to grow at 35 °C and even at 40 °C has a slow growth, confirming that the species is tolerant to high temperatures [43]. The data confirmed what reported in literature for *P. litorale* [33,44]. In other papers, where different strains were tested, 35 °C was reported as the maximum growth limit for *P. vexans* and 38 °C was reported as the optimal temperature for *P. helicoides* [31,45]. All of the tested strains showed an optimal growth at temperatures, which are in accordance with what has been observed in the soil of the orchards. In a monitoring performed in kiwifruit orchard during 2019, the average soil temperature measured during summer, when the KVDS symptoms occurred, was 23.2 ± 1.3 °C, whereas the average air temperature was 23.4 ± 4.5 °C.

The test to evaluate the tolerance of the species to different pH in vitro, showed their ability to grow at all the tested pH, ranging from 5.0 to 8.0. The maximum growth (*p* ≤ 0.05) was observed at pH 8.0 for *P. vexans*, at pH 6.5, 7.5 and 8.0 for *P. litorale* and at 5.0 and 5.5 for *P. helicoides*, whereas *P. chamaehyphon* seems the least influenced species by pH, where the highest growth was recorded in the range from 5.5 to 8.0. The average radial growth rate was similar for the species *P. vexans*, *P. litorale* and *P. chamaehyphon*, whereas *P. helicoides* showed the highest radial growth. For all the species under investigation, results showed that pH had less effect on growth than temperature, as previously shown by Cantrell and Dowler [45] for *P. vexans*. It should be noted that the effects of pH on fungal growth are complex, as an initial pH may affect growth, and subsequently growth can affect pH through release of metabolites into the growing medium. The values of pH and temperatures of the substrates recorded during pathogenicity tests were around the optimal values for the growth of the species of *Phytopythium* tested.

## 5. Conclusions

The present study demonstrates the strong role of oomycetes in the establishment and development of KVDS. The presence of different species of oomycetes suggests that the oomycete component of the soil microbiota present in the soil is involved in the development of KVDS, and not only a single species is involved in this complex syndrome. The isolated species have a relatively high optimal and maximum temperature for growth in soil, and they could be favoured by the average increase in soil temperatures during summer, associated with global warming. Waterlogging could exert a double effect, both on stressing the plant with a temporary anoxia and on favouring the release of infective zoospores by oomycetes. Further studies should investigate the complex interactions between kiwifruit, the oomycete species, the soil environment, and the effect of different management strategies in the field. Moreover, a study of the soil and rhizosphere microbiome could help to clarify the changes occurring in the soil microbiota of kiwifruit orchards.

## Figures and Tables

**Table 1:** Strain name, molecular identification, cultivar, year of isolation and GenBank accession numbers of the *Phytopythium* spp. strains isolated from kiwifruit roots in this study.

Strain *	Species	Cultivar	Year of Isolation	GenBank Accession Numbers **		
				ITS	LSU	COI
PH1	*Phytopythium vexans*	Hayward	2016	OL891590	OL957094	ON228590
**PH6**	*Phytopythium chamaehyphon*	Hayward	2016	OL891528	OL957144	ON228528
PH3	*Phytopythium vexans*	Hayward	2016	MN510425 *	**MN510427**	**MN510423**
PH2	*Phytopythium vexans*	Hayward	2016	OL891591	OL957095	ON228591
GBI	*Phytopythium vexans*	Hayward	2016	OL891578	OL957096	ON228578
4SRE1	*Phytopythium vexans*	Hayward	2016	OL891579	OL957097	ON228579
4SRE2	*Phytopythium vexans*	Hayward	2016	OL891580	OL957098	ON228580
4SBE_2C1	*Phytopythium vexans*	Hayward	2016	OL891581	OL957099	ON228581
4SBE_C2A	*Phytopythium litorale*	Hayward	2016	OL891534	OL957153	ON228534
4SBE_C2B	*Phytopythium litorale*	Hayward	2016	OL891536	OL957154	ON228536
4SBE_C2D	*Phytopythium litorale*	Hayward	2016	OL891533	OL957155	ON228533
4SBE_1C1	*Phytopythium vexans*	Hayward	2016	OL891587	OL957100	ON228587
4SBE_C1A	*Phytopythium vexans*	Hayward	2016	OL891586	OL957101	ON228586
4SBE_C2C	*Phytopythium litorale*	Hayward	2016	OL891535	OL957162	ON228535
4SBE_4C2	*Phytopythium vexans*	Hayward	2016	OL891588	OL957102	ON228588
4SBE_3C2	*Phytopythium vexans*	Hayward	2016	OL891589	OL957103	ON228589
4/16_DRE	*Phytopythium chamaehyphon*	Hayward	2016	OL891529	OL957145	ON228529
5/16_CBE	*Phytopythium vexans*	Hayward	2016	OL891582	OL957104	ON228582
6/16_BRE	*Phytopythium vexans*	Soreli	2016	OL891583	OL957105	ON228583
11/16_AB1	*Phytopythium chamaehyphon*	Hayward	2016	OL891531	OL957146	ON228531
12/16_DR2	*Phytopythium vexans*	Hayward	2016	**MN510426**	**MN510428**	**MN510424**
12/16_CR1	*Phytopythium vexans*	Hayward	2016	OL891584	OL957106	ON228584
12/16_AB1	*Phytopythium vexans*	Hayward	2016	OL891585	OL957107	ON228585
13/16_DR2	*Phytopythium vexans*	Hayward	2016	OL891564	OL957108	ON228564
13/16_DR3	*Phytopythium vexans*	Hayward	2016	OL891565	OL957109	ON228565
10/16_BR	*Phytopythium chamaehyphon*	Hayward	2016	OL891530	OL957147	ON228530
PP1	*Phytopythium vexans*	Hayward	2018	OL891571	OL957110	ON228571
PP2	*Phytopythium vexans*	Hayward	2018	OL891572	OL957111	ON228572
PP3	*Phytopythium vexans*	Hayward	2018	OL891573	OL957112	ON228573
PP4	*Phytopythium vexans*	Hayward	2018	OL891574	OL957113	ON228574
PP5	*Phytopythium vexans*	Hayward	2018	OL891575	OL957114	ON228575
PP6	*Phytopythium vexans*	Hayward	2018	OL891576	OL957115	ON228576
PP8	*Phytopythium vexans*	Hayward	2018	OL891577	OL957116	ON228577
**PPA**	*Phytopythium vexans*	Hayward	2018	OL891566	OL957117	ON228566
PPC	*Phytopythium vexans*	Hayward	2018	OL891567	OL957118	ON228567
PPD	*Phytopythium vexans*	Hayward	2018	OL891568	OL957119	ON228568
PPE	*Phytopythium vexans*	Hayward	2018	OL891569	OL957120	ON228569
PPF	*Phytopythium vexans*	Hayward	2018	OL891570	OL957121	ON228570
**CA1**	*Phytopythium helicoides*	Hayward	2019	OL891523	OL957151	ON228523
**CA2**	*Phytopythium helicoides*	Hayward	2019	OL891524	OL957152	ON228524
**CA3**	*Phytopythium chamaehyphon*	Hayward	2019	OL891525	OL957148	ON228525
CA4	*Phytopythium chamaehyphon*	Hayward	2019	OL891526	OL957149	ON228526
CA5	*Phytopythium chamaehyphon*	Hayward	2019	OL891527	OL957150	ON228527
**R1A**	*Phytopythium vexans*	Hayward	2019	OL891543	OL957122	ON228543
R1B	*Phytopythium vexans*	Hayward	2019	OL891544	OL957123	ON228544
R1C	*Phytopythium vexans*	Hayward	2019	OL891545	OL957124	ON228545
R1D	*Phytopythium vexans*	Hayward	2019	OL891546	OL957125	ON228546
R1E	*Phytopythium vexans*	Hayward	2019	OL891547	OL957126	ON228547
R1F	*Phytopythium vexans*	Hayward	2019	OL891548	OL957127	ON228548
R1G	*Phytopythium vexans*	Hayward	2019	OL891549	OL957128	ON228549
R1H	*Phytopythium vexans*	Hayward	2019	OL891550	OL957129	ON228550
**R3A**	*Phytopythium litorale*	Hayward	2019	OL891532	OL957156	ON228532
T4A	*Phytopythium vexans*	Hayward	2019	OL891562	OL957130	ON228562
T4B	*Phytopythium vexans*	Hayward	2019	OL891563	OL957131	ON228563
R7A	*Phytopythium vexans*	Hayward	2019	OL891551	OL957132	ON228551
R7B	*Phytopythium vexans*	Hayward	2019	OL891552	OL957133	ON228552
R7C	*Phytopythium vexans*	Hayward	2019	OL891553	OL957134	ON228553
R7D	*Phytopythium vexans*	Hayward	2019	OL891554	OL957135	ON228554
R7E	*Phytopythium vexans*	Hayward	2019	OL891555	OL957136	ON228555
R7F	*Phytopythium vexans*	Hayward	2019	OL891556	OL957137	ON228556
P8A	*Phytopythium vexans*	Soreli	2019	OL891557	OL957138	ON228557
P8B	*Phytopythium vexans*	Soreli	2019	OL891558	OL957139	ON228558
P8D	*Phytopythium vexans*	Soreli	2019	OL891559	OL957140	ON228559
P8E	*Phytopythium vexans*	Soreli	2019	OL891560	OL957141	ON228560
P8F	*Phytopythium vexans*	Soreli	2019	OL891561	OL957142	ON228561
**P8G**	*Phytopythium litorale*	Soreli	2019	OL891537	OL957157	ON228537
P8H	*Phytopythium litorale*	Soreli	2019	OL891538	OL957158	ON228538
P8I	*Phytopythium litorale*	Soreli	2019	OL891539	OL957159	ON228539
R10A	*Phytopythium litorale*	Dong Hong	2019	OL891540	OL957160	ON228540
R10B	*Phytopythium litorale*	Dong Hong	2019	OL891541	OL957161	ON228541
RE3	*Phytopythium vexans*	Hayward	2019	OL891542	OL957143	ON228542

* Strains in bold are used for morphological observations and in vitro tests. ** Sequences in bold are from Prencipe et al. [10].

**Table 2:** Results of disease severity (DS) on 1-year-old plants of *A. chinensis* var. *deliciosa* ‘Hayward’ plants at 12 and 26 dpi. Values in the same column followed by the same letter are not statistically different by Tukey test (*p* ≤ 0.05).

	DS 12 dpi			DS 26 dpi		
Treatment	Mean ± SD		Tukey Test	Mean ± SD		Tukey Test
*P. vexans* strain PP1	1.50 ± 0.50		bc	2.17 ± 0.58		b
*P. chamaehyphon* strain PH6	1.00 ± 0.50		ab	2.67 ± 0.29		bc
*P. helicoides* strain CA2	3.67 ± 0.58		d	4.00 ± 0.00		e
*P. litorale* strain R3A	2.17 ± 0.76		c	3.17 ± 0.76		cd
Positive control (infected soil)	3.50 ± 0.87		d	3.83 ± 0.29		de
Negative control (healthy soil)	0.00	-	a	0.00	-	a

**Table 3:** Dry weight (g) of roots sampled from 1-year-old plants of *A. chinensis* var. *deliciosa* ‘Hayward’ plants after 26 dpi. Values in the same column followed by the same letter are not statistically different by Tukey test (*p* ≤ 0.05).

Treatment	Mean ± SD	Tukey Test
*P. vexans* strain PP1	23.94 ± 9.16	b
*P. chamaehyphon* strain PH6	26.06 ± 2.77	b
*P. helicoides* strain CA2	7.35 ± 0.76	a
*P. litorale* strain R3A	17.13 ± 0.90	ab
Positive control (infected soil)	13.43 ± 2.91	a
Negative control (healthy soil)	40.05 ± 8.32	c

## Data Availability

See Section 2 Table 1.

## Supplementary Materials

The following supporting information can be downloaded at: https://www.mdpi.com/article/10.3390/microorganisms11010216/s1. Table S1: Strain name and information about orchards from where the strains were isolated (geographical location, geographical coordinates and orchard number); Table S2: List of species, strain designation and accession numbers for ITS and LSU regions and COI gene used for the phylogeny of *Phytopythium* spp. isolated from kiwifruit roots in this study.
