# Supplementary material for: Characterization of Phytopythium Species Involved in the Establishment and Development of Kiwifruit Vine Decline Syndrome
Source: Microorganisms. 2023 Jan 15;11(1):216. doi: 10.3390/microorganisms11010216 (PMC9862930; doi:10.3390/microorganisms11010216)
Supplement: Supplementary file 1 [file microorganisms-11-00216-s001.zip › microorganisms-2097359-supplementary.pdf]

Supplementary Table S1 - Strain name and information about orchards from where the strains were isolated (geographical location, geographical coordinates and orchard number).

Strain	Geographical location	Geographical coordinates		Orchard number
PH1	Alice Castello (VC)	45.341391	8.063265	1
PH6	Alice Castello (VC)			
PH3	Alice Castello (VC)			
PH2	Alice Castello (VC)			
GBI	Lagnasco (CN)	44.646031	7.536226	2
4SRE1	Lagnasco (CN)	44.64273	7.5318	3
4SRE2	Lagnasco (CN)			
4SBE_2C1	Lagnasco (CN)			
4SBE_C2A	Lagnasco (CN)			
4SBE_C2B	Lagnasco (CN)			
4SBE_C2D	Lagnasco (CN)			
4SBE_1C1	Lagnasco (CN)			
4SBE_C1A	Lagnasco (CN)			
4SBE_C2C	Lagnasco (CN)			
4SBE_4C2	Lagnasco (CN)			
4SBE_3C2	Lagnasco (CN)			
4/16_DRE	Costigliole Saluzzo (CN)	44.580377	7.52696	4
5/16_CBE	Saluzzo (CN)	44.637117	7.510636	5
6/16_BRE	Lagnasco (CN)	44.631046	7.569694	6
11/16_AB1	Manta (CN)	44.621428	7.50211	7
12/16_DR2	Lagnasco (CN)	44.631889	7.551037	8
12/16_CR1	Lagnasco (CN)			
12/16_AB1	Lagnasco (CN)			
13/16_DR2	Lagnasco (CN)	44.631889	7.542089	9
13/16_DR3	Lagnasco (CN)			
10/16_BR	Saluzzo (CN)	44.631051	7.500237	10
PP1	Revello (CN)	44.655846	7.417273	11
PP2	Revello (CN)			
PP3	Revello (CN)			
PP4	Revello (CN)			
PP5	Revello (CN)			
PP6	Revello (CN)			
PP8	Revello (CN)			
PPA	Manta (CN)	44.610421	7.50515	12
PPC	Manta (CN)			
PPD	Manta (CN)			
PPE	Manta (CN)			
PPF	Manta (CN)			
CA1	Verzuolo (CN)	44.59772	7.511737	13
CA2	Verzuolo (CN)			
CA3	Verzuolo (CN)			
CA4	Verzuolo (CN)			

Strain	Geographical location	Geographical coordinates		Orchard number
CA5	Verzuolo (CN)			
R1A	Manta (CN)	44.61569	7.541091	14
R1B	Manta (CN)			
R1C	Manta (CN)			
R1D	Manta (CN)			
R1E	Manta (CN)			
R1F	Manta (CN)			
R1G	Manta (CN)			
R1H	Manta (CN)			
R3A	Manta (CN)			
T4A	Manta (CN)			
T4B	Manta (CN)			
R7A	Saluzzo (CN)	44.64629	7.532261	15
R7B	Saluzzo (CN)			
R7C	Saluzzo (CN)			
R7D	Saluzzo (CN)			
R7E	Saluzzo (CN)			
R7F	Saluzzo (CN)			
P8A	Lagnasco (CN)	44.639418	7.584365	16
P8B	Lagnasco (CN)			
P8D	Lagnasco (CN)			
P8E	Lagnasco (CN)			
P8F	Lagnasco (CN)			
P8G	Lagnasco (CN)			
P8H	Lagnasco (CN)			
P8I	Lagnasco (CN)			
R10A	Scarnafigi (CN)	44.660261	7.547556	17
R10B	Scarnafigi (CN)			
RE3	Saluzzo (CN)	44.647014	7.551447	18

Supplementary Table S2 - List of species, strain designation and accession numbers for ITS and LSU regions and COI gene used for the phylogeny of *Phytopythium* spp. isolated from kiwifruit roots in this study.

		Accession numbers		
**Species**	**Strain designation**	**ITS**	**LSU**	**COI**
Phytopythium aichiense	CBS 137195	AB948197	AB948194	AB948191
Phytopythium boreale	CBS551.88	AY598662	AB690596	HQ708419
Phytopythium carbonicum	CBS112544	HQ643373	AB948196	HQ708420
Phytopythium chamaehyphon	CBS259.30	AY598666	AY598666	HQ708421
Phytopythium citrinum	CBS119171	HQ643375	AB948195	HQ708422
Phytopythium delawarense	CBS123040	EU339312	AB690598	KF853240
Phytopythium dogmae	USTCMS 4101	MF353170	MF373431	MF359559
Phytopythium fagopyri	CBS 293.35	AB690617	AB690590	AB690641
Phytopythium helicoides	CBS286.31	AY598665	HQ665186	HQ708430
Phytopythium iriomotense	CBS137104	AB690629	AB690607	AB690659
Phytopythium kandeliae	CBS113.91	HQ643133	HQ665079	HQ708206
Phytopythium leanoi	USTCMS 4102	MF353169	MF373430	MF359558
Phytopythium litorale	CBS118360	HQ643386	HQ665082	HQ708433
Phytopythium megacarpum	CBS 112351	HQ643388	AB690584	AB690635
Phytopythium mercuriale	CBS122443	AB690614	AB690585	AB690636
Phytopythium mirpurense	CBS124523	KJ831613	not available	KJ831612
Phytopythium montanum	CBS111349	AB725883	AB690586	AB690637
Phytopythium nanjingense	Chen 218	MF459636	not available	MF459633
Phytopythium oedochilum	CBS292.37	AB690619	HQ665191	AB690646
Phytopythium ostracodes	CBS768.73	AY598663	AB690587	AB690638
Phytopythium palingenes	CCIBt 3981	KR092139	KR092143	KT897702
Phytopythium sindhum	DAOM 238986	HQ643396	HQ665309	HQ708443
Phytopythium vexans	CBS119.80	HQ643400	HQ665090	HQ708447
Phytopythium vexans	NBRC107442	AB690626	AB690604	AB690656
Phytopythium vexans	NBRC107393	AB690630	AB690608	AB690660
Phytopythium vexans	NBRC107380	AB690627	AB690605	AB690657
Phytopythium vexans	NBRC107381	AB690628	AB690606	AB690658
Phytopythium vexans	NBRC107397	AB690610	AB690581	AB690632
Pythium ultimum	CBS 398.51	AY598657	HQ665227	HQ708906
